# Mesenchymal stem cell-released oncolytic virus: an innovative strategy for cancer treatment

**DOI:** 10.1186/s12964-022-01012-0

**Published:** 2023-02-24

**Authors:** Nadia Ghasemi Darestani, Anna I. Gilmanova, Moaed E. Al-Gazally, Angelina O. Zekiy, Mohammad Javed Ansari, Rahman S. Zabibah, Mohammed Abed Jawad, Saif A. J. Al-Shalah, Jasur Alimdjanovich Rizaev, Yasir S. Alnassar, Naseer Mihdi Mohammed, Yasser Fakri Mustafa, Mohammad Darvishi, Reza Akhavan-Sigari

**Affiliations:** 1 Isfahan University of Medical Sciences, Isfahan, Iran; 2 College of Medicine, University of Al-Ameed, Karbala, Iraq; 3 Department of Prosthetic Dentistry of the I.M. Sechenov First Moscow State Medical University (Sechenov University), Moscow, Russian Federation; 4 Department of Pharmaceutics, College of Pharmacy, Prince Sattam Bin Abdulaziz University, Al-Kharj, Saudi Arabia; 5 Medical Laboratory Technology Department, College of Medical Technology, The Islamic University, Najaf, Iraq; 6 Al-Nisour University College, Baghdad, Iraq; 7 Medical Laboratories Techniques Department, Al-Mustaqbal University College, Babylon, Iraq; 8 Department of Public Health and Healthcare Management, Rector, Samarkand State Medical University, Samarkand, Uzbekistan; 9 The University of Mashreq, Baghdad, Iraq; 10 Department of Pharmacy, Mazaya University College, Nasiriyah, Dhi Qar Iraq; 11 Department of Pharmaceutical Chemistry, College of Pharmacy, University of Mosul, Mosul, 41001 Iraq; 12 Department of Aerospace and Subaquatic Medicine, Infectious Diseases and Tropical Medicine Research Center (IDTMRC), AJA University of Medical Sciences, Tehran, Iran; 13 Department of Neurosurgery, University Medical Center, Tuebingen, Germany; 14 Department of Health Care Management and Clinical Research, Collegium Humanum Warsaw Management University, Warsaw, Poland

**Keywords:** Oncolytic virus, Mesenchymal stem cell, Cancer treatment, Oncolytic virotherapy, Cellular carriers

## Abstract

Oncolytic viruses (OVs) infect, multiply, and finally remove tumor cells selectively, causing no damage to normal cells in the process. Because of their specific features, such as, the ability to induce immunogenic cell death and to contain curative transgenes in their genomes, OVs have attracted attention as candidates to be utilized in cooperation with immunotherapies for cancer treatment. This treatment takes advantage of most tumor cells' inherent tendency to be infected by certain OVs and both innate and adaptive immune responses are elicited by OV infection and oncolysis. OVs can also modulate tumor microenvironment and boost anti-tumor immune responses. Mesenchymal stem cells (MSC) are gathering interest as promising anti-cancer treatments with the ability to address a wide range of cancers. MSCs exhibit tumor-trophic migration characteristics, allowing them to be used as delivery vehicles for successful, targeted treatment of isolated tumors and metastatic malignancies. Preclinical and clinical research were reviewed in this study to discuss using MSC-released OVs as a novel method for the treatment of cancer.

Video Abstract

**Supplementary Information:**

The online version contains supplementary material available at 10.1186/s12964-022-01012-0.

## Introduction

In recent years, early diagnosis of some cancer types, along with the development of cancer-specific treatments, has led to an increase in cancer patients' survival rates [1]. However, the short half-life of several cancer-specific medications, restricted distribution to particular tumor types, and negative impacts on healthy tissues are important barriers to treatment. Actually, the primary goal of cancer treatment is to develop anticancer medications that effectively target malignant cells while preserving healthy tissue [2, 3]. A few instances of metastatic cancer have been effectively treated using traditional methods [4]. As a result, developing a novel therapeutic approach to inhibit metastasis is crucial, especially given the issues with current cancer treatment strategies, such as drug resistance and systemic side effects [5, 6].

Beyond the capacity of some viruses to mediate oncogenesis and their use in the development of immunotherapies, such as the cytomegalovirus (CMV) gliomagenesis and its implications in the development of CMV-specific adoptive T cell immunotherapies, viruses themselves can be used as therapeutic agents to target tumor cells [7]. In this way, oncolytic viruses (OVs) are defined as naturally occurring or genetically manipulated viruses that exclusively replicate and grow in tumor cells and kill them while sparing normal cells [8, 9]. Oncolytic viral therapy is a new strategy of cancer therapy that has shown promise in preclinical and clinical trials [10, 11]. Altered mutants of human viruses, wild-type animal viruses that are cytotoxic to human cancer cells, and live virus vaccines are among the viruses used in this therapy. Adenovirus, measles virus, reovirus, herpes simplex virus, vesicular stomatitis, Newcastle disease virus, vaccinia virus, and poliovirus are some of these viruses [12, 13]. OVs have the ability to directly lyse cancer cells but this is not the sole advantage of them; it is now well acknowledged that one of the most essential aspects of virotherapy is the cytotoxic immune response they can trigger or reactivate in patients, which results in therapeutic responses [14, 15] and was shown in glioblastoma, B cell malignancy, metastatic melanoma, and liver cancer [16–20]. Indeed, multiple investigators have reported on the possible use of OVs for cancer treatment, with a demonstration of long-term prognosis [21]. A variety of factors, including viral elimination by the immune system and viral uptake by tissues and organs, can influence viral effectiveness in reaching cancerous tissues [22, 23, 24]. To boost treatment efficacy, effective carrier vehicles are essential for delivering OVs to tumor sites. Adult stem or progenitor cells have been extracted from a variety of tissues, including the brain, heart, and kidney, and have shown promise in treating a variety of diseases [25–27]. In both in vitro and many murine cancer models, unmodified MSC has been demonstrated to have anti-tumor activities. This is due to antitumor substances generated by MSCs, which limit the growth of cancer cells including glioma, melanoma, lung cancer, hepatoma, and breast cancer [28–33]. Furthermore, MSCs have been utilized as carriers because of their known tumor-specific homing ability, which allows for the virus's safe transportation and releases on the tumor site [34–37]. Using MSCs might be a method to enhance the quantity of oncolytic virus given to patients while reducing side effects and avoiding direct tumor injections [38]. Altogether, in this paper we will review the features of MSCs and OVs as well as their activities against tumors, and also discuss challenges in using this strategy as an innovative cancer treatment.

## The features of the MSC-based delivery of OV

As a novel cancer treatment method, virotherapy offers various benefits, including the likely absence of cross-resistance with traditional treatments and the ability to promote tumor elimination through a variety of pathways. In addition, MSCs are suitable carriers for anticancer viruses since they can home to tumor sites [39–41, 42], are easy to isolate and develop in vitro, and have strong metabolic activity, which is necessary for viral replication [34, 43].

## MSCs as OV carriers

The majority of preclinical research has discovered efficient features for MSCs as OV carriers [44–46] and this strategy might be an effective way to promote oncolytic virotherapy efficiency [47–54]. Indeed, MSCs have been shown to be one of the best alternatives for OV chaperoning in the treatment of cancer due to their tumor-homing, inherent anticancer capacities, OVs preservation from neutralizing antibodies, and viral distribution to the tumor site via the Trojan horse strategy [53, 55, 56].

In a series of studies, MSC has been discovered to migrate to the area of injury, ischemia, and tumor sites via chemotaxis [57]. Although the mechanisms by which MSC migrate across the endothelium or to targeting sites remain unclear, extensive research has demonstrated that MSC migration is regulated by the cytokine/receptor pairs, such as SDF-1/CXCR4, SCF/c-Kit, HGF/c-Met, VEGF/VEGFR, PDGF/PDGFR, MCP-1/CCR2, and HMGB1/RAGE [58]. In the TME, many immune cells, as well as cancer cells, release soluble molecules that can directly influence MSC chemotaxis to injured tissues. The oxidative state, vascularization, and inflammatory condition of the tumor can all influence MSC migration efficiency at these locations [59]. It has been shown that interleukin-6 (IL-6) can promotes MSC tropism to cancer sites [60] and also this movement is IL-8-dependent in glioma [61]. Tumors can also recruit MSCs from other tissues, such as bone marrow (BM-MSCs) and adipose tissue (AD-MSCs), and promote their engraftment into the TME through inflammatory signals [62–64]. Local variables including hypoxia, cytokines, and Toll-like receptor (TLR) ligands stimulate recruited MSCs to multiply and express growth factors that enhance tissue regeneration at the site of injury [65]. Hepatic carcinoma [66], breast cancer [67], and glioma have all been demonstrated to attract MSCs [68].

This therapeutic strategy, in addition to offering substantial site-specificity, avoids potential issues associated with biological drug half-life limitations, as drug release might be tailored to be constant [43]. Moreover, it's hard to manage effective concentrations of anti-tumor drugs near the tumor for long periods of time [69]. For instance, in the case of brain tumors, the failure of substances to cross the blood–brain barrier is a concern. The use of MSCs as cellular delivery vehicles has been proposed as a novel approach to addressing these obstacles, allowing for a more precise and long-lasting therapeutic response than standard delivery methods would ordinarily allow [2]. Another unique aspect of employing MSCs as OV carriers is that they may function as biological manufacturers for viral genome replication, enhancing virus titer. This implies that a low initial dose of OVs for loading into MSCs is sufficient to deliver a high viral dose to tumor microenvironment. However, the specifics of OVs replication within MSCs remain unknown [70, 71]. As a result, when OVs are carried by MSCs, they leverage MSCs' natural affinity to reach tumor sites, improving OVs homing and promoting oncolysis.

### Immunosuppressive activities of MSCs

Multiple studies revealed that MSCs have anti-inflammatory and immunosuppressive properties, so that, these cells generate and release a number of soluble cytokines, such as IL-6, IL-10, TGF-β1, heme oxygenase-1(HO-1), inducible nitric oxide synthase (iNOS), and indoleamine-2-dioxygenase-3(IDO) [72]. These cytokines are essential in immunosuppression and inhibiting B lymphocyte maturation and restricting their capacity to produce immunoglobulin [73–75], inhibiting the secretion of cytokines by helper T cells, reducing the cytotoxic actions of effector T lymphocytes [76], decreasing NK cell proliferation, cytotoxicity, and cytokine generation [77]. Furthermore, in a study on diabetic nephropathy in rats, it was shown that MSCs suppressed CD103+ DCs and CD68+ CD11c+ macrophages in the kidneys and alleviate renal injury [78–81]. They are also able to inhibit the differentiation of CD14+ monocytes and CD34+ progenitor cells into mature DCs [82], restrict DC differentiation and function [83], and thereby increase CD4 + CD25 + FOXP3+  T lymphocytes (Treg) development and generation of other regulatory immune subtypes, including CD8+ CD28−T lymphocytes [84, 85], IL-10-producing B lymphocytes [86], and IL-10-producing DCs [87]. These actions are essential MSC characteristics for inhibiting local inflammation during virotherapy and permitting the oncolytic virus to replicate and destroy cancer cells without immune constraint. [88].

### Anti-tumor effects of MSCs

Several investigations have indicated the specific homing of oncolytic virus-loaded MSCs to tumor xenografts and consequent infection of tumor cells, results in diminished tumor sizes and a considerable improvement in the survival rates of treated animals [89–95]. MSCs enhance the proliferation of some tumor cell lines in vivo but not others. Variations in tumor types, MSC preparations, duration, and quantity of MSC delivery may cause differences in the functional role of MSCs in tumor growth [96]. Indeed, MSCs are thought to limit tumor development by interrupting the cell cycle, reducing proliferation, inhibiting the PI3K/AKT pathway, and expressing suppressor genes [97, 98]. In a breast cancer metastasis mouse model, the umbilical cord derived MSCs (UC-MSC) and AD-MSCs were administered, and it was shown that they could prevent lung metastasis and slow tumor development by cleavage of the poly (ADP-ribose) polymerase (PARP) and caspase-3, which could then trigger apoptosis [99]. Another study showed that murine bone marrow MSCs had a cytotoxic impact on the tumor in a melanoma murine model through the production of reactive oxygen species when in interact with endothelial cells located at the capillaries [100]. As a result, the tumor development was delayed and the endothelial cells undergo apoptosis. Nevertheless, the MSCs' cytotoxic effects were only apparent when they were implanted in large quantities [101]. In addition, in a mouse model of Kaposi sarcoma, human MSCs (hMSCs) administered intravenously (iv.) were found to home to carcinogenesis sites and potently decrease tumorigenesis. Cell contact via E-cadherin and Akt inhibition were essential for the suppression of the sarcoma cells proliferation [102]. MSCs have been also discovered to display anti-angiogenic properties in vitro and in melanoma murine models [101]. Moreover, AD-MSCs have both (pro- and anti-cancer) capabilities in breast [103] and prostate cancer [104].

## Mechanisms of oncolytic virotherapy

Various mechanisms of action are used by oncolytic viruses. Selective replication inside tumor cells leads to a direct lytic impact on tumor cells and the development of a systemic anti-tumor immune response. Depending on the origin and kind of cancer cell, the viral vector's properties, and the interactions between the virus, TME, and host immune response, the proportional involvement of different mechanisms could differ [105]. Indeed, Oncolytic virotherapy depends on a balance of antiviral mechanisms that kill the virus and pro-immune mechanisms that detect cellular epitopes, TAAs, and neoantigens from virus-infected tumor cells [106]. In addition to their anti-tumor properties, OVs stimulate antiviral immunity against viral antigens from the resulting infection, which is a critical player during OV-based treatments due to its capability to create an advantageous microenvironment for the immune system's activity against specific cancer cell indicators [107, 108].

The two major methods by which OVs destroy tumors are direct cell death and the activation of anti-tumor immunity [109]. The first strategy employs the virus's biological life cycle; oncolytic viruses may destroy cancer cells infected with them by direct virus-induced cytotoxicity, which is regulated by a variety of cytotoxic immune activation pathways. Following cell proliferation and lysis, virions infect surrounding cells and repeat the lytic cycle, enabling the treatment to self-amplifying at the site of need [110]. This cycle repeats until the virus's replication is reduced or the number of vulnerable host cells is decreased [111]. On the other hand, OVs are one of the most well-known immunogenic cell death inducers, and they're more probably to be type II stimulators than type I stimulators [112]. Although the precise mechanisms by which oncological viruses operate are elusive, it is suspected that they function by regulating the infected cancer cell's molecular cell death machinery. The majority of cancer cells may be resistant to apoptosis [113] and according to researchers, they have several apoptosis evasion mechanisms but they may be driven to die by non-apoptotic processes. Recent studies demonstrated that OVs can induce immunogenic cell death (ICD) in cancer cells through immunogenic apoptosis, necroptosis, necrosis, autophagic cell death, and pyroptosis, which exposes calreticulin and HSPs to the cell surface and/or releases ATP, HMGB1, uric acid, and other DAMPs as well as PAMPs as danger signals, along with tumor-associated antigens (TAAs), to activate dendritic cells and elicit effective antitumor immunity [114, 115]. However, it was recently shown that dendritic cell-mediated anti-tumor immunity is compromised by cancer cells undergoing ferroptosis [116]. To respond to the tumor antigens, antigen-presenting cells (APCs) catch TAAs and neoantigens released by tumor cells, and then activate tumor-specific T lymphocytes [117, 118]. On the surface of tumor cells infected with OVs, there are virus-specific antigens, which help in their elimination by antiviral T lymphocytes [119]. As a result, even if the virus does not reproduce well, OVs can trigger an antitumor immune response [106]. Additionally, the breakdown of the tumor's immunological tolerance is acknowledged as a major feature of OV's method of action and can destroy the tumor [120, 121].

Additionally, the OV will infect tumor cells and control protein production, increasing viral macromolecule creation while also stimulating the expression and detection of danger signals. Indeed, these are the results of some signaling pathways that end in the release of DAMPs such as heat shock proteins (HSPs), calreticulin, uric acid, and ATP and cytokines including, interferons (IFNs), tumor necrosis factor-α (TNF-α) and IL-12, all of which help to improve immune responses [105, 106, 122, 123]. Pathogen-associated molecular patterns (PAMPs), such as nucleic acids, proteins, and viral capsid elements, are also released as a result of virus-induced tumor cell death [124, 125, 126, 127]. These compounds help counteract the immunosuppressive condition of the TME by promoting the migration and activation of macrophages, NK, DC, and tumor-specific cytotoxic T cells [128–130]. The TME is made up of tumor cells, local or penetrated non-transformed cells (e.g., cancer-associated fibroblasts, vascular endothelial cells, immune recruitment cells), secretory substances, and the extracellular matrix. In fact, this microenvironment is generally immunosuppressive and tumors produce soluble immunosuppressive agents such as nitric oxide and cytokines including IL-10 and TGF-β, resulting in active suppression of efficient anti-tumoral immune response [106, 131, 132]. Furthermore, Tregs and myeloid-derived suppressor cells (MDSCs) are directed to the TME, where they use the acquired immune response pathway's potential to detect and eliminate tumor cells [106, 132, 133].

The immunostimulatory versus immunosuppressive behavior of the TME is controlled by cytokines and immune cells [107]. OVs enable proinflammatory cytokines to enter the TME, establishing a suitable condition for DC activation. By activating antigen presentation pathways in tumors, OVs stimulate DCs to detect tumor antigens [134, 135]. In this environment, OVs can outperform various evasion methods. For example, by treating with oncolytic reovirus, an ovarian cancer cell line expressed more MHC class I and other molecules related to antigen processing, such as the transporter associated with antigen processing (TAP) and β2-microglobulin (β2M). This action enhanced DC maturation, which resulted in adaptive immunological responses driven by CD8 T cells [136]. Moreover, in a murine model treated with an engineered adenovirus, many splenic CD11c CD8 DCs were found an tumor-infiltrating plasmacytoid DCs revealed a mature phenotype capable of priming tumor-specific cytotoxic T cell activities [137]. Furthermore, multiple studies have shown that other OVs, including vaccinia virus [138], measles virus [139], and HSV [140], can improve DC antigen presentation, which is commonly associated with enhanced expression of costimulatory/activation components such CD80, CD86, and MHC II.

The tumor-associated macrophage population is an important modulator of the immunostimulatory versus immunosuppressive behavior of the TME. Anti-viral and anti-cancer responses are related to M1 pro-inflammatory macrophages, whereas metastasis, angiogenesis, and inhibition of anti-cancer and anti-viral responses are related to M2 immunosuppressive macrophages [141–143]. As shown by oncolytic paramyxovirus infection of macrophages, OVs act as potent immunological triggers and are useful to modify the phenotypic activities of macrophages [144]. OVs are able to provide an inflammatory environment that encourages macrophage infiltration and activation. For instance, in a xenograft colorectal cancer model, it was shown that treatment with the oncolytic vaccinia virus GLV-1h68 caused a considerable increase in proinflammatory cytokines like IL-3, IL-6, IFN-, and CXCL10. It causes boosting the recruitment of proinflammatory macrophages to the tumor site [145]. Similar to this, a triple combined treatment including oncolytic HSV boosted macrophage recruitment and M1-like polarization, which helped to eliminate glioblastoma [146].

**Fig. 1:**
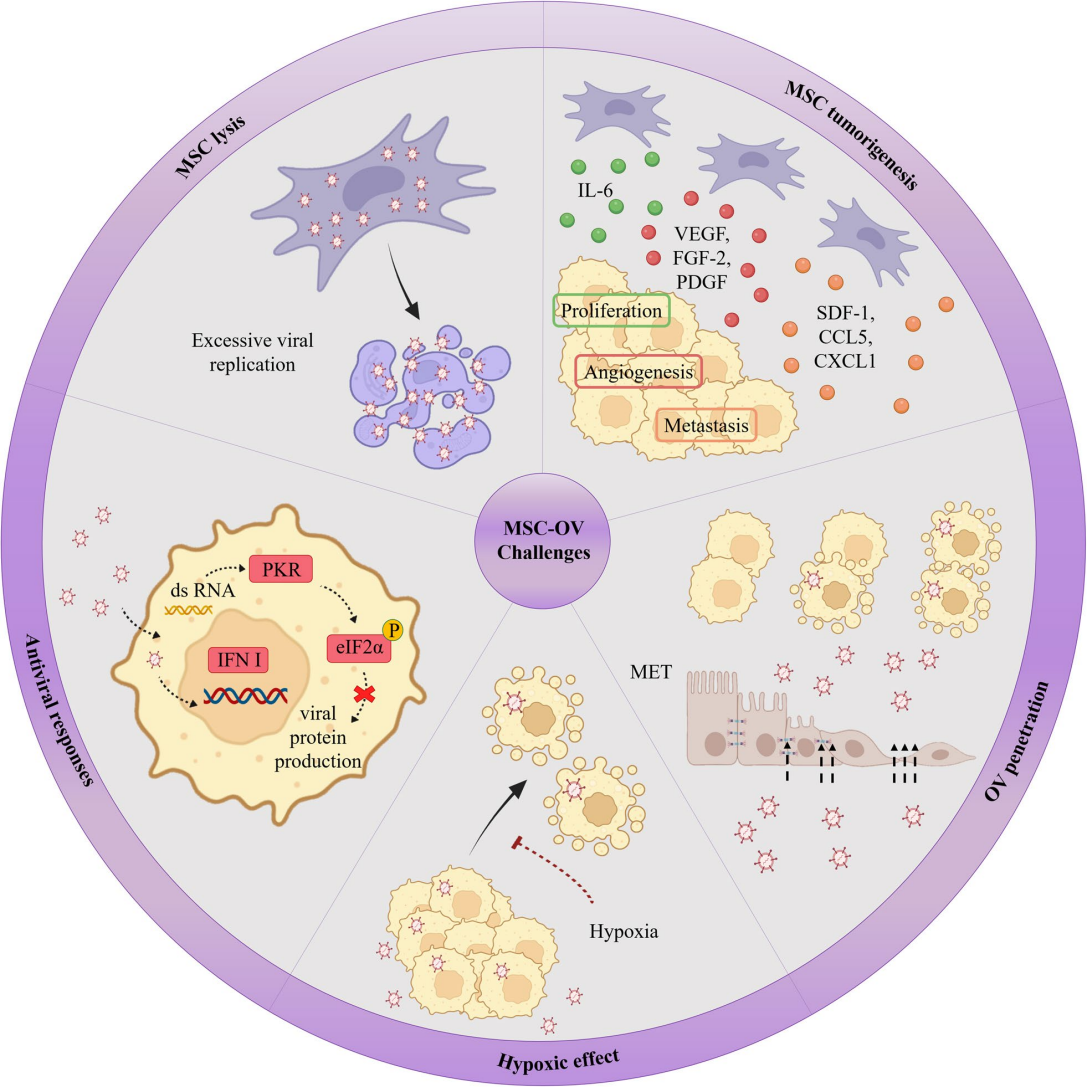
Mesenchymal stem cell-based delivery of oncolytic virus challenges. MSCs might stimulate angiogenesis, tumor cell proliferation, and metastasis by release large numbers of cytokines and growth factors, including VEGF, FGF-2, βFGF, PDGF, IL-8, IL-6, CXCL1, CCL5, and SDF-1. MET during metastasis tighten epithelial connections and make therapy challenging. Hypoxic circumstances have been observed to decrease viral proliferation and lytic capacity. Type I IFN hinder intra-tumoral spread of the OVs, moreover, infection with double-stranded RNA leads to PKR activation in the cell. Excessive viral replication may result in premature MSC lysis and reduce the efficiency. *VEGF* vascular endothelial growth factor, *FGF-2* fibroblast growth factor 2, *PDGF* platelet-derived growth factor, *IL* interleukin, *CXCL* c-x-c motif chemokine ligand 1, *CCL5* c–c motif chemokine ligand, *SDF-1* stromal cell-derived factor 1, *MET* mesenchymal-to-epithelial transitions, *IFN I* type I interferon, *PKR* RNA-dependent protein kinase, *eIF2α* eukaryotic initiation factor 2 α

The typical antiviral response from healthy cells, which can limit OV reproduction and disseminate directly, is one of the earliest defenses to OVs. Type I IFN is one of the key drivers of this activity (IFN-a and IFN-b) [141, 142, 147, 148]. Type I IFN plays a significant part in anti-cancer responses by triggering immune cells inside the TME, including NK cells and CD8+ T cells, and pro-inflammatory cytokines, in addition to regulating the anti-viral condition [148, 149]. Because of its regulatory impact on NK cells and CD8+ T cells, Type I IFN enhances anti-tumor immune responses. Activated NK cells release type II interferon (IFN-γ), which suppresses angiogenesis, promotes apoptosis, and stimulates the immune system (by activating MHC class II in DCs, macrophage phagocytic activity, and CD8+ T cell responses) [107]. Type I IFN can also increase MHC class I expression in DCs, as well as co-stimulatory molecules (CD40, CD86) and the Th1 polarized response [141, 142] (Fig. 1). Furthermore, the capability of OV-infected tumor cells to produce type I IFN in each tumor site, as well as the potency by which certain OVs stimulate type I IFN signaling, varies significantly among OVs, and these facts all influence the OV's potential to activate the acquired immune system against viral and cancer antigens [142]. To achieve the appropriate balance between anti-viral and anti-cancer immunity, more research into the impact of each OV is required.

Angiogenesis is a cancer characteristic that involves the supply of nutrients and oxygen to tumor cells to enhance tumorigenesis [150, 151]. Several OVs have been demonstrated to have anti-angiogenic properties by inducing an acute disturbance of the tumor vasculature [152–154]. For instance, it has been found that the oncolytic vaccinia virus inhibits tumor angiogenesis, limits blood supply to tumor cells, and ultimately leads to hypoxia through impacting vascular cells [155, 156–158]. In addition, OVs can destroy uninfected cancer cells by damaging tumor blood vessels and enhancing particular antitumor immune responses. The transgene-encoded proteins produced by modified viruses can assist oncolytic viruses in killing uninfected cancer cells [159].

## MSC-based delivery of oncolytic adenovirus (oAds)

Since the oAds replication could be limited to malignant cells, these viruses are being evaluated in clinical trials for the treatment of several malignancies [160–162]. (Table 1) In fact, after the oAds replicates in tumor cells, the cell is lysed and more infectious virions are produced, infecting and lysing adjacent tumor cells and consequently inducing endogenous tumor immune responses which has therapeutic benefits [163]. It has been revealed that MSCs are finally lysed by oAd replication which avoids any negative side effects related to stem cell viability in vivo [59, 164]. In addition, multiple investigations of individuals with ovarian cancer, melanoma, soft tissue or primary bone sarcoma, and other neoplasms have revealed that oAds have a high safety profile [10, 160, 161, 162, 165–169]. For instance, Garcia-Castro and colleagues demonstrated the safety and effectiveness of delivering numerous doses of autologous MSCs infected with ICOVIR-5, which is an optimized oncolytic adenovirus, to four children with metastatic neuroblastoma who had failed to respond to standard therapy [170]. An in vivo study also revealed that infected MSCs transport the combination of ICOVIR15 and Ad.iC9 to lung tumor sites effectively and specifically increase overall survival and tumor control [50]. Furthermore, in Stoff Khalili et al. research, hMSCs were utilized as intermediate vehicles for conditional replication oncolytic adenoviruses (CRAds) to target breast cancer and reduced the development of pulmonary metastasis, most likely due to viral replication in the hMSCs [90]. Pulmonary metastasis in patients with breast cancer is common characteristic [171, 172, 173] and is associated with lethal complications in these patients [174, 175]. Furthermore, when CRAd-loaded MSC was given intravenously to mice with solid ovarian cancer, it had a considerably stronger anticancer impact and a prolonged survival time than CRAd delivered directly [89]. Moreover, it has been shown that hepatocellular carcinoma (HCC)-targeted oAd can be loaded into MSCs successfully by viral capsid modification, and oAds induce significant cancer-specific death pathways through active viral reproduction in the MSC driver. Indeed, oAd-loaded MSCs improve both oAd and MSC safety features by reducing oAd hepatic sequestration and hepatotoxicity while improving MSC clearance through viral proliferation [59]. Also, Rincon et al. revealed that MSCs carrying the oncolytic adenovirus ICOVIR5 were therapeutically effective in treating lung cancer in mice by inhibiting tumor development and encouraging T cell migration to the tumors [176]. Similar to this, MSCs infected with the oncolytic adenovirus CRAd5/F11 prevented tumor growth in a colorectal cancer subcutaneous mouse xenograft model [177]. In the other study Kaczorowski et al. [178] removed the antiapoptotic gene E1B19K from oAd and replaced it with the cell death ligand TRAIL gene. After intravenous injection of infected MSCs, adenoviral capsid protein was found in tumor xenograft tissue but not in healthy tissue, indicating tumor-specific migration [178]. Similarly, direct in vivo therapy was associated with a significantly decreased tumor size, lower Ki67 and CD24 expression, and increased caspase activation [160]. Chastkofsky et al. evaluated the potential of MSC for OV delivery for the treatment of diffuse intrinsic pontine glioma (DIPG. They utilized the survivin promoter for conditional replication of OV. Their results revealed that cells and tumors have increased expression of survivin and cell surface proteins which provide effective OV entry and replication in DIPG cells and result in more prolonged survival [179]. To elaborate, survivin is an anti-apoptotic factor that facilitates the viability and survival of cells and has been identified as a target in the treatment of several autoimmune diseases and cancers [180, 181].

**Table 1:** Mesenchymal stem cell-released oncolytic virus for cancer treatment

OVs type	Other name	Specimen	MSC source	Cancer type	Ref
oAds	ICOVIR‐5	Human	MSC	Metastatic neuroblastoma	[170]
	oADs	Human	hMSC	Malignant gliomas	[91]
	rAd.DCN	Mice	UC-MSCs	Breast cancer lung metastasis	[182]
		Mice	hUCB-MSCs	Glioma	[183]
	HCC-oAd-WNTi	Athymic nude mice	BM-MSC	HCC	[59]
	oADs	Mice	BM-MSC	NSCLC	[50]
	CRAd Ad5/3.CXCR4	SCID mice	hMSC	Metastatic breast cancer	[90]
	CRAd	Mice	MSC	Ovarian cancer	[89]
	oADs	Mice	BM-MSC	PDA	[178]
	Δ24-RGD	Athymic mice	BM-MSC	GBM	[92]
oHSV	oHSV, R-LM249	Nude, NSG mice	FM-MSC	Lung and brain metastases	[49]
	oHSV	SCID, C57BL6 mice	hMSC	Brain metastatic melanomas	[184]
	oHSV	SCID mice	BM-MSC	GBM	[185]
oMV	oMV	SCID mice	BM-MSC	HCC	[95]
	oMV	Mice	AD-MSC	Ovarian cancer	[53]
	oMV	SCID mice	BM-hMSC	ALL	[186]
MYXV		Human	AD-MSCs	Malignant brain tumor	[187]
		Immune competent mice model	BM-MSCs	Pulmonary Melanoma	[188]
		Mice	AD-MSCs	Murine pancreatic adenocarcinoma	[189]
		Immunocompetent mice	AD-MSCs	Murine pancreatic adenocarcinoma	[190]
oncolytic reovirus	ReoT3D	Mice	AD-MSCs	Colorectal cancer	[191]
	ReoT3D	Cell line	AD-MSCs	TC-1 Cell line	[192]
		Immunocompromised AML mouse model	hUC-MSCs	AML	[193]

*oAds* oncolytic adenovirus, *oMV* oncolytic measles virus, *oHSV* herpes simplex virus, *SCID* severe combined immunodeficiency, *hMSC* human MSC, *BM-MSC* bone marrow-drived MSC, *FM-MSC* fetal membrane MSC, *AD-MSC* adipose tissue-derived MSC, *HU-MSC* human umbilical cord-derived MSC, *HCC* human hepatocellular carcinoma. *ALL* acute lymphoblastic leukemia, *GBM* glioblastoma multiforme, *NSCLC* non-small-cell lung carcinoma, *PDA* pancreatic ductal adenocarcinoma, *CRAD* conditional replication oncolytic adenoviruses

In a mouse model of breast cancer with pulmonary metastases, Zhang et al. investigated the therapeutic effects of MSCs infused with OAd expressing decorin, a naturally occurring inhibitor of TGF-β signaling. They demonstrated that MSCs boosted the therapeutic benefits of oncolytic adenoviral administration and dissemination in tumor tissues [182]. Since the oncolytic adenovirus replicates in MSCs, it is essential to balance MSC viability with viral load in order to get the best therapeutic outcome. Recently, Zhang et al. developed an all-in-one Tet-on system that capable of controlling the reproduction of OAd. The new OAd expressing Endostatin and/or IL-24 was then introduced into hUCB-MSCs for the treatment of glioma. Their findings showed that this new OAd was capable of killing glioma cells with high efficiency while sparing healthy cells [183].

Interestingly, Barlabé et al. equipped the OAdv with a therapeutic transgene to provide the strongest anticancer effects. They demonstrated that, when menstrual blood-derived mesenchymal stem cells (MenSCs) are combined with ICOVIR15-cBiTE, an OAdv producing an EGFR-targeting bispecific T-cell engager (cBiTE), the antitumor effectiveness is increased in comparison to MenSCs loaded with the unarmed virus ICOVIR15 [194]. Combining MSC-delivered OV with prodrug activation is another strategy to achieve optimum effectiveness. Under these circumstances, MSCs might convert the simultaneously delivered prodrug into cytotoxic metabolites, causing oncolysis and inhibiting tumor development without being hazardous to the host's essential organs [195].

Adoptive cell treatments for solid tumors have a significant challenge due to the immunosuppressive TME [196]. Oncolytic immunotherapy using modified OAd by infecting tumor cells, may disrupt the TME [197]. It was recently revealed that lung cancers could be successfully treated in animal models using a combination of cell carrier-delivered OAd and chimeric antigen receptor (CAR-T) cells. A binary vector including an OAd and a helper-dependent Ad (HDAd; combinatorial Ad vector (Cad)) that expresses checkpoint PD-L1 blocker and IL-12 has been demonstrated by this work to be systemically delivered by MSCs [198]. The immune checkpoint ligand PD-L1 has been found to be overexpressed in a variety of solid tumors [199]. PD-L1 binds to its receptor PD-1, and blocking it has shown therapeutic potential in cancer therapy [200]. However, when PD-L1 blockade is combined with optimal dose IL-12 delivery, it induces a synergistic effect of enhancing anti-tumor immunity in cancer patients [201].

## MSC-based delivery of oncolytic herpes simplex virus (oHSV)

The oHSV has been evaluated extensively in combination with MSCs and has demonstrated promising outcomes in the treatment of gliomas, metastatic melanomas, breast, and ovarian cancers, whether administered systemic [49, 184] or locally [185]. For example, in order to investigate efficiency of oHSV in immune-deficient and immune-competent murine models of melanoma brain metastasis, Du et al. utilized MSCs as cellular vehicles for oHSV and revealed that MSC-oHSV effectively detects metastatic tumor deposits in the brain, inhibits brain tumor development, and extends survival [184]. Furthermore, in a clinically relevant glioma model, Duebgen M, et al. infused hMSCs with oHSV and revealed that they successfully generated oHSV progeny, cause tumor resection and resistance and extended average lifespan in mice [185]. Indeed, one of the most important alternatives for GBM treatment is the oHSV, which is a naturally neurotrophic factor [8, 202, 203]. In addition, it has been shown that MSCs from various origins can be infected and loaded with a HER2-retargeted oncolytic HSV and the metastatic burden in the brain was shown to be reduced by more than half in NSG mice, which showed significant suppression of breast cancer brain metastases [49] (Table 1).

## MSC-based delivery of oncolytic measles virus (oMV)

The oMV exhibits significant anticancer potential and is being studied as an innovative tumor therapy in a number of Phase I clinical studies [204, 205]. Indeed, oMV reproduction, protein expression, syncytia formation, and oncolysis have been reported in vivo, in a variety of human tumor xenografts, such as hematologic malignancies like lymphoma [206, 207] and myeloma [208], as well as solid tumors including ovarian cancer [209], glioblastoma [210], hepatocellular carcinoma [211], prostate cancer [212, 213], breast cancer [214], cervical cancer [215], and gastric cancer [216, 217]. On the other hand, MSCs have been demonstrated to be efficient carriers of attenuated oMV to ovarian tumors [94] and HCC [95]. For instance, in the hepatocellular carcinoma and murine model, two groups investigated intravenous single delivery of oMV-loaded BM-MSCs. Both measles antibody-naive and passively-immunized SCID mice showed a considerable decrease in tumor development when treated with oMV-infected BM-hMSCs [95]. Moreover, in an orthotopic ovarian cancer therapy model, treated mice with AD-MSCs as drivers of oMV survived longer in comparison to mice treated with a naked virus or uninfected MSC [53]. In addition, in a xenograft model, BM-hMSCs were shown to successfully delivering OMVs to precursor B-lineage acute lymphoblastic leukaemia (ALL) cells. Ex vivo loading of oMV into BM-MSCs was effective and oMV was replicated intracellularly without toxicity [186].

Early research [208, 210, 212, 218, 219, 220] reported an increase in apoptotic markers in infected cells, whereas Lampe et al. demonstrated viral cells die even when apoptosis inhibitors are used, indicating that alternative cell death pathways are involved [221]. Donnelly, OG et al. revealed that Measles virus causes immunogenic cell death (ICD) in human melanoma[222].

## MSC-based delivery of oncolytic myxoma virus (MYXY)

In order to cure a malignant brain tumor in mice, human adipose-derived MSCs (AD-MSCs) have been coupled with MYXV, which expresses the reporter gene green fluorescent protein (GFP) [187]. This study established the potential of AD-MSCs to produce new viral particles as well as the ability of infected cells to adhere to tumors when administered intravenously. As a consequence, mice treated with the OV-loaded MSCs had a much higher survival rate than mice treated with MSCs alone.

BM-MSCs have also shown to be permissive to MYXV replication [188]. Furthermore, the scientists showed improved antitumor activity in an immunological competent lung melanoma model following intravenous treatment, in comparison to MYXV monotherapy, employing an IL-15-armed MYXV to infect BM-MSCs. Only animals treated with the virus without cell carriers showed an enhanced proportion of circulating NK cells, suggesting that MSCs may have inhibited the immune system from recognizing the infection. Finally, tumors from animals treated with MYXV-IL-15-loaded MSCs showed an increase in pro-inflammatory cytokines, programmed cell death protein 1 (PD-1)/programmed death-ligand 1 (PD-L1), and infiltration of effector T cells, suggesting a potential antitumor immune response as a result of the therapy.

In a separate investigation, recombinant MYXV, which encodes murine LIGHT, also known as tumor necrosis factor ligand superfamily member 14 (TNFSF14), was used to infect AD-MSCs ex vivo. Results from this study showed that mice with orthotopically induced pancreatic ductal adenocarcinoma (PDAC) had increased trafficking into the pancreas compared to tumor-free animals, which led to the extended survival of the treated PDAC-seeded animals and the increased expression of important adaptive immune response markers. Administered IP and pre-loaded ADSCs with transgene-armed MYXV really enable more efficient oncolytic virus ferrying to PDAC sites and promote better tumor regression [189]. Additionally, it has been shown that combining the pre-loaded LIGHT (TNFSF14)-Armed Myxoma virus with Gemcitabine (as an antimetabolite) may be a potential strategy to enhance the therapeutic benefits of vMyx-LIGHT/ADSCs against PDAC in vivo [190] (Table 1).

## MSC-based delivery of oncolytic Reovirus

A wide range of human malignancies, including acute myeloid leukemia, have been treated with reovirus, a naturally occurring OV [223]. The potential use of an oncolytic reovirus created by Reolysin®, (pelareorep; wild-type reovirus; Serotype 3 Dearing; Oncolytics Biotech Inc.), for the treatment of various tumor cells was examined in several clinical trials [224]. Reolysin®, a novel systemically administered promising anti-cancer drug for pancreatic, ovarian, and malignant glioma tumors, received FDA approval in 2015 [225, 226]. The oncolytic reovirus's anti-cancer effect against the glioblastoma multiforme (GBM) cell line may be enhanced by AD-MSCs, which are a vulnerable host for the virus [227]. In vitro cancer treatment using MSCs that have been infected with reovirus type-3 Dearing (T3D) has also been examined. According to the study's findings, reovirus-infected AD-MSCs in TC-1 cells produce more NO and TNF-α than normal while producing less TGF-β1 and IL-10. Additionally, TC-1 cells were co-cultured with infected AD-MSCs, which greatly enhanced apoptosis when compared to the control [192].

In a different study, Babaei et al. examined the anti-tumor potential of adipose-derived mesenchymal stem cells (AD-MSCs) as a novel delivery system for the Dearing strain of reovirus (ReoT3D), which has the highest capacity to eradicate cancer cells among other strains in a murine model of colorectal cancer. They found that ReoT3D and MSCs together were more effective for therapy than ReoT3D and MSCs alone [191]. Wang et al. demonstrated that Human UC-MSCs harboring reovirus display antitumor effectiveness impacts for AML in the presence of Nabs via increasing CXCL10 production from hUC-MSCs [193] (Table 1).

## Clinical studies using MSC-OV

Only a few clinical trials have used MSC-OV to treat cancer patients (Table 2). Autologous MSCs loaded with oAd (ICOVIR) or CELYVIR were employed in the first-in-human trial for the treatment of pediatric refractory metastatic neuroblastoma (NCT01844661) [38].

**Table 2:** Clinical trials on the MSC-OV utilization in cancer patients

Status	Interventions	Conditions	Number Enrolled	Identifier
Completed	Biological: CELYVIR	Children Solid Tumors Metastases	20	NCT01844661
Recruiting	Biological: Oncolytic Adenovirus Ad5-DNX-2401 Procedure: Therapeutic Conventional Surgery	IDH1 wt Allele Recurrent Anaplastic Astrocytoma Recurrent Glioblastoma	36	NCT03896568
Recruiting	Other: Laboratory Biomarker Analysis Procedure: Mesenchymal Stem Cell Transplantation Biological: Oncolytic Measles Virus Encoding Thyroidal Sodium Iodide Symporter	Fallopian Tube Clear Cell Adenocarcinoma Fallopian Tube Endometrioid Adenocarcinoma Fallopian Tube Mucinous Adenocarcinoma	57	NCT02068794
Not yet recruiting	Biological: AloCelyvir	Uveal Melanoma, Metastatic	16	NCT05047276

*oAds* oncolytic adenovirus, *DNX-2401* Tasadenoturev

Another phase I/II clinical trial examined the adverse effects and optimal dosage of MSCs infected with the oncolytic measles virus that encodes the thyroidal sodium iodide symporter (MV-NIS) and how effectively it treats patients with recurrent ovarian, primary peritoneal, or fallopian tube cancer (NCT02068794). Also, another phase I trial, evaluated the optimum dose and side effects of the oncolytic adenovirus DNX-2401-loaded hBM-MSCs in treating patients with recurrent high-grade glioma through intra-arterial administration (NCT03896568). Also, the safety and efficacy parameters of AloCELYVIR in Metastatic uveal melanoma patients with hepatic metastases were examined in phase I/II clinical trial (NCT05047276) (Table 2).

## Challenges

**Fig. 2:**
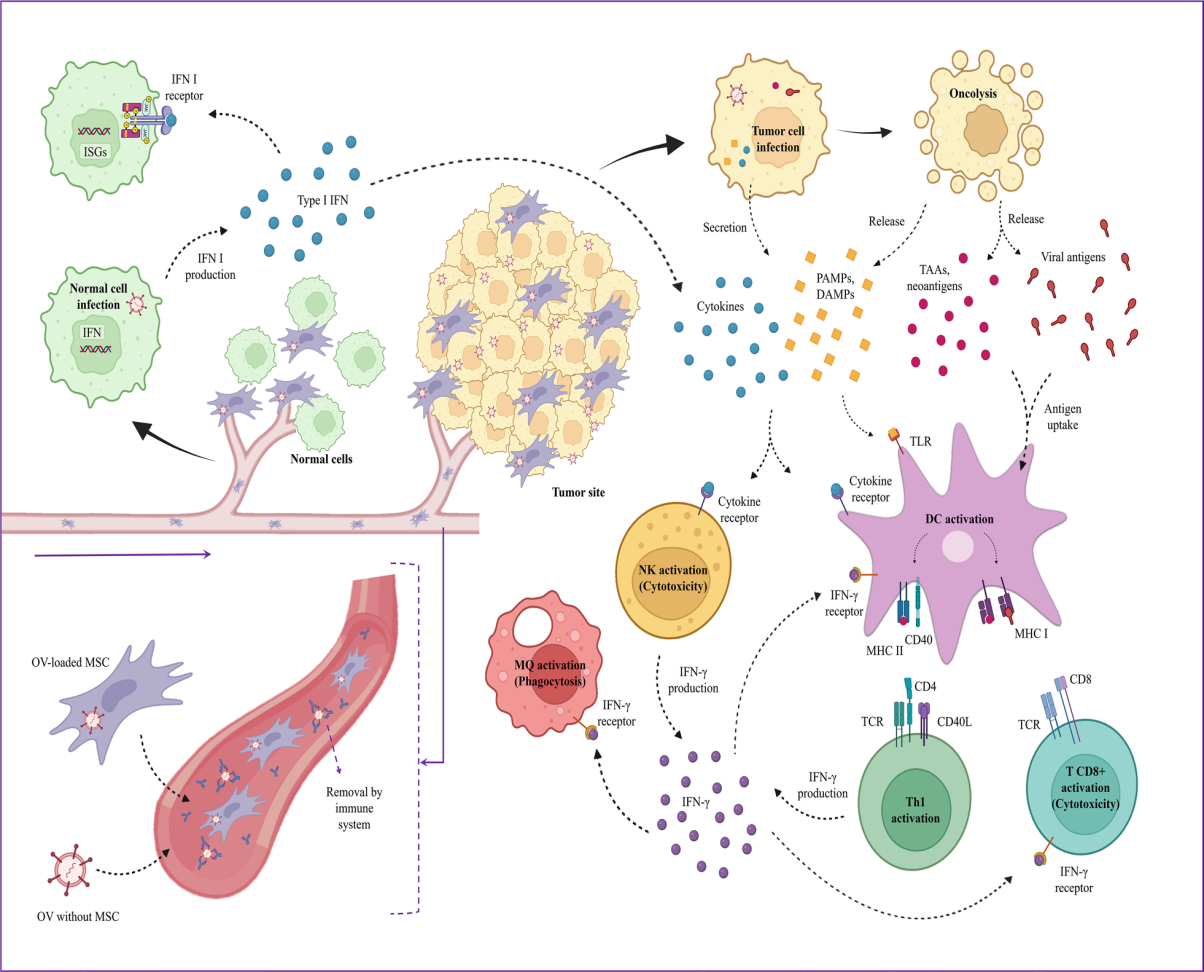
MSCs feature as OVs carriers and mechanisms of MSC-released OVs in cancer treatment. OVs are maintained by MSCs from immune system responses. MSCs migrate to the tumor site via chemotaxis. There are two major methods by which OVs destroy tumors are direct cell death and the activation of anti-tumor immunity. Tumor cells secret and release DAMPs such as HSPs, calreticulin, uric acid, and ATP and cytokines including, IFNs, TNF-α and IL-12, and PAMPs, such as nucleic acids, proteins, and viral capsid elements as a result of OVs infection and oncolysis. These compounds help counteract the immunosuppressive condition of the TME by promoting the migration and activation of MQs, NK cells, DCs, and tumor-specific cytotoxic T cells. Normal cells antiviral response also includes type I IFN which can play a significant part in anti-cancer responses by triggering immune cells inside the TME. *DAMPs* damage-associated molecular patterns, *HSPs* heat shock proteins, *ATP* adenosine triphosphate, *IFNs* interferons, *TNF-α* tumor necrosis factor-α, *PAMPs* pathogen-associated molecular patterns, *MQs* macrophages, *NK cells* natural killer cells, *DCs* dendritic cells, *TME* tumor microenvironment

Despite progress in our understanding of MSC-based OV delivery, there are still significant challenges ahead, which raises questions and concerns that are debatable and scientists have proposed a number of solutions to these problems, which are detailed below (Fig. 2).

### MSC tumorigenesis

The interaction of tumor cells with healthy cells and the stroma in the TME is getting important since these interactions have a role in critical stages of tumor progression including angiogenesis, immunomodulatory, metastasis, invasion, and apoptotic resistance [113, 228, 229]. Moreover, reports have argued that MSCs enhance or suppress tumor growth and metastasis through a variety of mechanisms, including the secretion of soluble molecules that trigger or repress innate and adaptive immune responses, activate or reduce angiogenesis, and sustain the cancer stem cell environment [230, 231, 232]. Indeed, MSCs can alter the rate of tumorigenesis depending on the circumstances [36, 233]. According to studies, MSCs release trophic factors that can boost tissue angiogenesis, cell proliferation, and cell survival [234–236]. For example, large numbers of cytokines and growth factors that stimulate angiogenesis, including VEGF, FGF-2, βFGF, PDGF, IL-8, IL-6, angiopoietin, and TGF, are released by MSCs and contribute to the development of tumor angiogenesis [237–239]. Additionally, cancer-associated fibroblasts (CAFs) actively encourage tumor angiogenesis by producing chemokines and cytokines such as IL-4, IL-8, IL-6, TNF, CXCL12, TGF, and VEGF which have anti-inflammatory and pro-angiogenic properties [240]. CAFs differentiation is enhanced by interactions between MSC and tumor cells [237, 241, 242]. Furthermore, MSCs stimulate epithelial-mesenchymal transition (EMT) by secreting growth factors and cytokines such as HGF, PDGF, EGF, and TGF, which cause the production of transcriptional regulators of EMT like Slug, Snail, Zeb1, and Twist [243, 244]. Evidence suggests that abnormal EMT enhances tumor metastasis, drug resistance, and tumor growth [245]. The epithelial cell-related proteins E-cadherin, ZO-1, and -catenin/plakoglobin are downregulated during the EMT while mesenchymal proteins including fibronectin, N-cadherin, smooth muscle actin, and vimentin are increased [71, 246]. In addition through the release of chemokines such as CXCL1, CCL5, CXCL5, CXCL8, and CXCL7 by MSCs, tumor cell migration to metastatic sites is accelerated [247, 248]. It has been also shown that MSCs release large amounts of CXCL12 (SDF-1), which control the invasion and migration of tumor cells that express CXCR4 [249, 250]. MSCs polarization in response to substances released by the tumor, is another explanation for the conflicting results, which either forces the cells into a tumor-promoting or suppressive action. Accordingly, an in vitro co-culture of MSC1 with several cancer cell lines reduced the growth of tumors, while the MSC2 co-culture had a contradictory outcome. Similarly, MSC1 treatment of tumors established in immune-competent mice reduced tumor growth and metastasis, whereas MSC2 treatment promoted tumor development and dissemination [159]. Indeed, inhibiting or activating certain MSC TLRs might be a promising approach for enhancing the anticancer effects of OV oncolysis by adding MSC immune-stimulatory features.

As a result, a greater understanding of the particular molecular processes behind these pro-tumorigenic actions is essential for further improving anti-cancer therapeutic approaches. Enhancing the therapeutic effects for cancer patients would be achievable through the reduction of MSC recruitment into tumor areas and the suppression of their tumor-supportive actions, particularly with the combination of other therapeutic methods, such as immunotherapy.

### MSC lysis

However, while viral replication within MSCs is a desirable feature, excessive viral replication may result in premature MSC lysis and reduce the overall efficiency [59]. It has been demonstrated that a large initial viral dosage reduces total MSC survival with just a little increase in overall viral production [59].

### Antiviral responses

Patients’ antiviral immune responses are a major concern, limiting the impact of oncolytic viruses [251, 252]. The patient's antiviral defense is triggered and recruited to limit virus reproduction and dissemination, resulting in viral elimination and the treatment impact being lost [106]. Antiviral cytokines, including various forms of IFN, are a barrier to an efficient anti-tumor response to OV because hinder intra-tumoral spread of the OVs [253]. Several studies have utilized histone deacetylase (HDAC) inhibitors to promote epigenetic changes and reduce antiviral cytokine responses in the TME to resolve this challenge [254–256]. Furthermore, infection with double-stranded RNA leads to PKR activation in the cell. PKR can phosphorylate the α-subunit of eukaryotic initiation factor 2 α (eIF2α) which keeps it inactive and prevents viral protein production [257, 258]. However, in vitro and in vivo studies have shown that Sunitinib inhibits the antiviral enzymes RNase L and PKR, which impedes antiviral innate immune responses [258].

### OV optimizing

When determining the ideal OV treatment method, inherent qualities should be regarded. Each OV family will have its genomic complexity, replication methods, lytic qualities, transgene packaging capacity, and immune response-inducing ability to activate anti-tumor immunity. Since different OVs have distinct tumor tropisms, it has been difficult to identify precise molecular biomarkers that predict particular anti-tumor efficacy for each OV [133, 259]. Moreover, optimizing the initial OVs loading dosage is a critical factor that improves loading efficacy and affects treatment effectiveness [260]. Additionally, the oncolytic virus lifecycle's time is an essential factor in the carrier cells' tumor-homing potential. Indeed, before the viral progeny is released, the delivery cell must concentrate in tumor sites to provide effective delivery of the therapeutic virus [261].

### Targeting methods

MSCs can be targeted using a variety of methods, such as physical, physiological, and biological ones that attempt to increase their density in a specific area [262]. Physical targeting is inserting cells directly into the area that requires therapy via surgical methods or guiding techniques like catheters or external magnets [263, 264, 265]. In addition, a different approach is to contain therapeutic cells in a matrix or devices that maintain cells in the transplant area [262]. For example, it has been demonstrated that MSC encapsulation in a biodegradable, synthetic extracellular matrix dramatically boosted their survival in the GBM excision cavity while permitting the production of anti-cancer proteins [185, 266, 267]. Another method employs physiological mechanisms like the systemic circulation to transfer the cells rather than active cell-mediated migration [262]. For instance, cells frequently become caught in the lungs' capillaries. To distribute MSC-mediated treatments to the lungs, this effect can be used [54, 90]. The MSCs trapping in the lung seems to be dependent on several factors, such as administration route and vessel size. Intravenous administration of MSCs results in an accumulation of cells in the lungs, which are then redistributed to the kidneys, spleen, and liver. Arterial injection bypasses the lungs, so MSCs are widely distributed throughout the rest of the body. There is insufficient systemic biodistribution during intramuscular, intraarticular and intradermal administration [268, 269]. In spite of the route of injection of MSCs [66], the size of the vessels are also play a key role in MSCs trapping in lung. Scherpfer et al. showed that the average size of MSCs is larger than the size of pulmonary capillaries. Therefore, large amounts of administered MSCs can become trapped in the capillaries of the lung and prevent access to other organs. In addition, vasodilator could be reduced lung localization [270].

Furthermore, biological targeting techniques have been developed to satisfy the demand for greater target stringency following systemic administration of MSCs, particularly when the disease to be treated is extensive, as in metastases [271, 272]. It includes evidence-based techniques aiming at enhancing MSC homing, binding selectivity to a target tissue, and persistence within the target environment [262]. Indeed, to regulate MSC homing potential, various approaches have been established, including altering the MSC culture conditions to promote the production of homing-related compounds, redesigning the cell membrane to increase homing, and adjusting the target tissue to better attract MSCs [273].

### OV penetration

Epithelial junctions function as an obstacle to the intracellular infiltration of OVs, particularly adenoviruses, in carcinomas [274]. Indeed, phenotype changes during metastasis, including EMT and later mesenchymal-to-epithelial transitions (MET), which tighten epithelial connections and make therapy challenging [275, 276]. Yumul et al. created epithelial junction openers (JO) by modifying Ad5Δ24. They found that oncolytic Ads that express JO had a substantially higher anti-tumor activity than unmodified viruses [274]. Moreover, the extracellular matrix (ECM) and cellular connections are significant barriers that are related to the spread and penetration of OVs. In fact, OVs must cross the complex ECM to reach tumor cells and lysis them [277]. Pre-treatment of cancer with collagenase [278] or co-administration of hyaluronidase with oncolytic adenoviruses [279] resulted in increased viral dissemination. Additionally, altering OVs to express matrix metalloproteinases-1 and -8 causes cancer-associated sulfated glycosaminoglycans to be degraded, resulting in improved viral dispersion and treatment efficiency [280]. Tumor cell apoptosis also promotes viral dissemination. For instance, Nagano et al. found that cytotoxic substances induced apoptosis and activated caspase-8, resulting in greater intratumoral uptake and anti-cancer effect of oncolytic HSV. They hypothesized that reducing or eliminating apoptotic tumor cells resulted in channel-like structures and empty areas, enabling oncolytic HSV to disseminate more easily [281].

### Hypoxic effect

Hypoxia is a characteristic of solid tumors that emerges throughout the formation and development of the tumor and has been demonstrated to have paradoxical impacts on OVs [282]. Hypoxic circumstances have been observed to decrease viral proliferation and lytic capacity without changing the expression of surface receptors [283, 284]. Because hypoxia may cause cell cycle arrest, this feature might influence the capability of oADs and other viruses that rely on cell cycle advancement to reproduce [284]. Clarke et al. created an oncolytic adenovirus in which the expression of the E1A gene is regulated by the hypoxia-response factor-containing promoter to counteract hypoxic suppression of viral reproduction and to get the benefits of hypoxic conditions for homing [285]. However, in 2009, two groups revealed that a hypoxic condition increases oncolytic HSV viral proliferation [286, 287]. This might be due to HSV's intrinsic affinity to low-oxygen cells or DNA damage caused by oxygen-derived free radicals, which promotes HSV reproduction [286].

### Treatment durability

Tumors frequently recur after great initial treatment results. Stem cell treatment using a single substance, like other chemotherapies, isn't always successful in removing tumors [288, 289]. As a result, a reasonable medication combination should be determined [290]. Many different combination therapies have been tried to see whether they can help with treatment persistence. For instance, irradiating cancer cells leads them to release molecules that promote MSC penetration across integral basement membranes, resulting in an increase in the amount of MSCs in cancers [291].

## Modification

In addition to their inherent potential to lyse cancer cells, OVs can be modified to improve their lytic activity. For example, adenoviruses expressing the herpes simplex virus-1 thymidine kinase (HSV-1 TK) under the osteocalcin promoter have been designed to target bone cancers. HSV-1 TK could convert thymidine analogs, such as ganciclovir into monophosphates, which stop DNA synthesis and trigger cell death By incorporating into the DNA of reproducing cells [292, 293, 294]. OVs have been modified to improve immune responses even further. Most transgenes are designed to induce an adaptive immune response against cancer antigens or to contribute to the treatment of immune cell-depleted malignancies. Including, cytokines, chemokines, inhibitory receptors, co-stimulatory receptors, bispecific cell engagers, immunological ligands, and combinations of any of these [133, 295, 296]. For instance, researchers have engineered oncolytic viruses which can express IL-2, IL-12, IL-15, IL-6, IL-21, IL-18, IL-24, and granulocyte–macrophage colony-stimulating factor (GM-CSF) and activate various aspects of the immune system [295, 296]. Moreover, the immunosuppressive TME might be altered by inserting an immune stimulatory chemical into OV genomes. The most often utilized example is GM-CSF, which has been inserted into OV genomes as an immune stimulatory molecule to promote the maturation and recruitment of APCs, particularly DCs, as well as the recruitment of tumor antigen-specific T cells and NK cells [109]. On the other hand, one aspect of transgenic-armed OVs is that immune activation can be delayed depending on the viral promoter that regulates the transgene or by controlling protein translation. To avoid an overly-rapid immune response, the expression of transgenes should be postponed until the viral oncolysis is at its maximum [297]. Additionally, the kind of transgenic and the number of transgenes that may be included in a single viral construct are both influenced by the type of virus. Unlike DNA viruses, which can handle more transgenes without harming replication, RNA viruses generally have a shorter genome and can only encode a restricted number of them [133]. Furthermore, a modified oncolytic adenovirus expressing the TRAIL gene was recently utilized to treat a mouse model of pancreatic ductal adenocarcinoma (PDAC), a malignant and lethal malignancy with a poor prognosis and few treatment options. The study revealed that in a PDAC animal model, AD-MSCs carrying TRAIL specifically homed to the cancer site and significantly slowed tumor growth, with no toxicity or adverse effects [178].

MSCs can be also genetically manipulated or preconditioned to increase their intrinsic features, such as improved migration, adhesion, and survival, as well as reduced premature aging. For example, to improve MSC migration, CXCR1, 4, and 7 were overexpressed which CXCR1 binds to IL-8 and CXCR4 and CXCR7 bind to SDF-1. Also for increasing MSC adhesion ability, MSCs were genetically engineered to express higher levels of integrin-linked kinase (ILK) [298].

## Conclusion and prospect

MSCs improve the anticancer efficacy of virotherapy in a variety of ways. Indeed, MSCs act as a reproduction site for OVs, allowing for the generation of more virions, which is advantageous for virotherapy. In addition, MSCs' tumor tropism and immunosuppressive activity enable the virus to specifically target the cancer site, increasing viral spread, and survival. MSCs, on the other hand, generate cytokines that attract immune cells to the TME, increasing the anticancer immune response. Moreover, oncolysis triggers the production of danger signal including TAAs and DAMPs/PAMPs, which stimulate local anticancer immune responses and alter the TME from immunosuppressive to immunostimulatory [45, 51].

Integrating MSCs with more effective OVs is a reasonable move towards enhancing therapeutic outcomes. Currently, there are four ongoing clinical trials using the OV-loaded MSCs for cancer therapy, which offer up a wide range of combinations with MSCs [299].

Altogether, further development of MSCs-OVs therapies may rely on a multifaceted strategy to select design parameters to improve the safety profile and efficacy of carrier cells, improve viral replication in MSCs, and establish patient eligibility criteria. For overcoming these obstacles some efforts have performed. For example, by manipulating the MSCs, it is feasible to enhance the clinical result. Polymers or other viral capsids might potentially be used to improve infectivity and viral replication [300, 301]. Also to control the adenovirus’s replication within MSCs, an all-in-one Tet-on system has been developed, which could help future studies to reach the optimum therapeutic effect of the oncolytic virus [183].

To summarize, although existing clinical trials will help to clarify the therapeutic efficacy of MSCs as OV cell carriers, further efforts should be undertaken to translate current viral and cellular preclinical achievements to the clinic, either as monotherapy or in combination with radiation, chemotherapy, or even immunotherapies.

## Data Availability

Not applicable.

## Abbreviations

OVs: Oncolytic viruses
TME: Tumor microenvironment
MSCs: Mesenchymal stem cells
UC-MSC: Umbilical cord derived MSCs
hMSCs: Human MSCs
BM-MSCs: Bone marrow MSCs
DAMPs: Damage-associated molecular patterns
PAMPs: Pathogen-associated molecular patterns
oAds: Oncolytic adenovirus
CRAds: Conditional replication oncolytic adenoviruses
oHSV: Herpes simplex virus
oMV: Oncolytic measles virus
EMT: Epithelial-mesenchymal transition
MET: Mesenchymal-epithelial transitions
